# Revealing the Effect of Synthesis Conditions on the Structural, Optical, and Antibacterial Properties of Cerium Oxide Nanoparticles

**DOI:** 10.3390/nano11102596

**Published:** 2021-10-01

**Authors:** Nicusor Fifere, Anton Airinei, Marius Dobromir, Liviu Sacarescu, Simona I. Dunca

**Affiliations:** 1 Petru Poni Institute of Macromolecular Chemistry, 41A Grigore Ghica Voda Alley, 700487 Iasi, Romania; airineia@icmpp.ro (A.A.); livius@icmpp.ro (L.S.); 2 Department of Exact and Natural Sciences, Institute of Interdisciplinary Research, Alexandru Ioan Cuza University of Iasi, 11 Carol I Blvd., 700506 Iasi, Romania; marius.dobromir@uaic.ro; 3 Department of Microbiology, Biology Faculty, Alexandru Ioan Cuza University of Iasi, 11 Carol I Blvd., 700506 Iasi, Romania; sdunca@uaic.ro

**Keywords:** cerium oxide nanoparticles, modified precipitation method, lattice parameters, XPS, antibacterial activity, optical properties

## Abstract

Cerium oxide nanoparticles were prepared by a precipitation method using Ce(IV) sulphate as precursor dispersed in glycerol with varying synthesis parameters such as temperature or precipitating agent. The structural and morphological characteristics of the obtained nanoparticles were investigated by X-ray diffraction, transmission electron microscopy, and diffuse reflectance spectroscopy. The crystallite size of the nanoparticles varied between 13 and 17 nm. The presence of Ce^3+^ and Ce^4+^ was proved by XPS data in the CeO_2_ samples and the conservation of the fluorite structure was evinced by X-ray diffractograms with a contraction of the lattice parameter, regardless of the size of the nanoparticle. From diffuse reflectance spectra, two band gap energy values for the direct transition were observed. Depending on the synthesis condition, the red shift of gap energy and the blue shift of Urbach energy with increasing content of Ce^3+^ were ascertained. The antibacterial tests revealed that the cerium oxide nanoparticles show good antimicrobial activity towards the common pathogens *Escherichia coli* and *Staphylococcus aureus*.

## 1. Introduction

The potential applications of cerium oxide nanoparticles are mainly determined by the existence of cerium ions in the two oxidation states, +3 and +4, in which Ce^4+^ ion has the predominant weight. The remarkable physico-chemical properties are emergent from the presence of Ce^3+^ ions in the crystalline network, without distorting the global structure of the CeO_2_ crystal. Some properties, such as quantum confinement as well as high surface-to-volume ratio, induced by the nanosized dimensions of the cerium oxide, together with the low redox potential of the Ce^4+^/Ce^3+^ couple, determine heterogeneous processes at the nanoparticle surface in contact with air or liquid. Industrial applications of the materials based on cerium oxide nanoparticles include UV protective materials [1], oxygen sensor and storage [2,3], catalysis [4,5], and solid oxide fuel cells [6,7]. Reversible redox reaction of the Ce^4+^/Ce^3+^ couple determines the pro- or antioxidant properties of cerium oxide nanomaterials with potential utilizations in medicine, playing a dual role as a cell protector against oxidative stress, but also as a sensitizer in cancer radiotherapy or in antibacterial applications [8,9].

The performance of cerium oxide nanoparticles, in different applications, is given by the ability of these materials to coordinate different species of oxygen at the surface level, but also by the efficiency of the redox switching process Ce^4+^ ↔ Ce^3+^. These two processes act synergistically in cerium oxide materials, leading to interesting properties that recommend them for the aforementioned applications. However, these properties are in close connection with different types of defects, especially oxygen vacancies, leading to structural asymmetries and sites with unbalanced charge or incomplete or unusual coordination. All these features cause the surface and deeper zones near the surface to have more energy and thus a higher reactivity in defective nanosized ceria structures such as CeO_2-γ_. Therefore, defect engineering has been addressed to control the surface chemistry of the ceria nanoparticles, making them suitable for the fundamental superficial physico-chemical processes required in various applications. In addition to other defects, oxygen vacancies play an essential role in the catalytic and antioxidant activity of nanoparticles, their presence being a direct consequence of the coexistence of both oxidation states of cerium, Ce^4+^ and Ce^3+^.

Various methods for the synthesis of cerium oxide nanoparticles have been approached: precipitation, sol-gel, hydrothermal, solvothermal, thermal hydrolysis, thermal decomposition, spray pyrolysis, ball milling vacuum deposition techniques, and so on [10]. In order to assure the generation of various defects in the cerium oxide nanostructures, doping procedures have generally been applied, especially with lower valence cations [11]. On the other hand, this method leads to the depreciation of the Ce^3+^ concentration, affecting the Ce^3+^ ↔ Ce^4+^ oxidation–reduction cycles. These are necessary to achieve high catalytic and antioxidant performances, so doping processes with such cations must be made carefully. In order to not affect such characteristics, it is of interest to introduce structural defects, implicitly oxygen vacancies, by modification the synthesis parameters, without using foreign metal doping. In this regard, Dowding et al. [12] were among the first to study the biomedical applications of cerium oxide nanomaterials with different contents of oxygen vacancies, obtained by changing the precipitating agent during the synthesis, leading to a tuned control of the surface ratio of the Ce^4+^/Ce^3+^ couple. He et al. [13] revealed the importance of activated faces of the nanoparticles by the presence of oxygen vacancies. In this sense, they synthesized nanoceria with different exposed faces, modifying the conditions of hydrothermal synthesis. Generally, at high temperature and low oxygen pressure, oxygen deficient cerium oxide, CeO_2-γ_, is formed [14] with a pronounced non-stoichiometry, but with a preserved fluorite structure. The appearance of oxygen vacancies is accompanied with the presence of reduced cerium ion Ce^3+^ in the lattice with a higher radius than Ce^4+^. Usually, this difference in the ionic radius induces an increase in the lattice parameter as the nanoparticle size decreases [15]. Despite this affirmation, the variation of the lattice parameter with grain size seems to be more complex and non-monotonic, depending on the route of synthesis [16].

In the majority of cases, the synthesis of cerium oxide nanoparticles is done starting from precursors consisting of salts of trivalent cerium. The main reactions of the formation of nanoceria consist of the hydrolysis–precipitation of trivalent cerium ions with the formation of cerium hydroxide, followed by its oxidation, and then the completion of the process with the dehydration reaction and the formation of cerium oxide. By modifying the reaction conditions as well as the nature of the precipitation agent, a wide range of cerium oxide nanoparticles can be obtained. There are very few studies on the synthesis of nanoceria from precursors consisting of tetravalent cerium ion salts. Even so, the majority of them refer to water-soluble salts in relatively large amounts, such as double salts of cerium and ammonium [17,18]. The difficulty is due to the very high Lewis acidity of Ce^4+^ ions, which, being strongly hydrated, lead to an acid hydrolysis reaction by complexing water molecules and HO^−^ groups according to the following reaction [19]:

$${\mathrm{Ce}}^{4+}+\left(x+y\right)H_{2}O↔{\left[\mathrm{Ce}{\left(H_{2}O\right)}_{x}{\left(\mathrm{OH}\right)}_{y}\right]}^{\left(4-y\right)+}+{\mathrm{yH}}^{+}$$

The stability of the neutral aqueous solution of the hydrated Ce^4+^ ions is very low due to the high possibility of the deprotonation–dehydration reaction, as follows [20]:

$${\left[\mathrm{Ce}{\left(H_{2}O\right)}_{x}{\left(\mathrm{OH}\right)}_{y}\right]}^{\left(4-y\right)+}\overset{\;H_{2}O\;}{\to }{\mathrm{CeO}}_{2}\cdot {\mathrm{nH}}_{2}O+{\mathrm{mH}}_{3}O^{+}$$

This leads to a colloidal suspension in water, consisting of a mixture of hydroxide and cerium (IV) oxide, making manipulation of the solution of Ce^4+^ ions harder than for the solution of Ce^3+^.

The aim of this paper was to develop the synthesis of nanoceria starting from a precursor almost insoluble in water at room temperature and neutral pH. Also, the structural and morphological characteristics of cerium oxide nanoparticles were discussed in connection with the modification of synthesis parameters and the evaluation of antibacterial properties of the resulting products. The synthesis was based on property of Ce^4+^ ion to complexate and oxidize polyols [21], but also on the switching ability of the redox couple Ce^4+^/Ce^3+^. The morphological characterization was conducted using XRD and TEM data to show the dimension of the grain and its shape, as well as the crystal structure. The defect generation following the variation of the synthesis parameters was discussed comparatively using XPS, UV-Vis absorption, and fluorescence spectroscopy. Regarding the antibacterial properties, Gram-positive and Gram-negative pathogenic bacteria were taken under consideration with nanoparticle concentration variation.

## 2. Materials and Methods

### 2.1. Materials

Cerium sulphate tetrahydrate Ce(SO)_4_·4H_2_O (>99%, Sigma-Aldrich, Saint Louis, MO, USA), sodium hydroxide (>98%, Sigma-Aldrich, Saint Louis, MO, USA), glycerol (99.5%, Sigma-Aldrich, Saint Louis, MO, USA), ammonia (25%, Chemical Company, Iasi, Romania), and hydrogen peroxide (30%, Chemical Company, Iasi, Romania) were utilized in the synthesis process and were of analytical grade without any further purification. Double deionized water was used for synthesis and washing.

### 2.2. Preparation of CeO_2_ Nanoparticles

In a typical procedure, CeO_2_ nanoparticles were synthetized via the precipitation procedure, taking cerium (IV) sulphate tetrahydrate as precursor material and an aqueous solution of sodium hydroxide as precipitating agent. In detail, 3 g of cerium salt was dispersed and dissolved in 60 mL of glycerol at 70 °C under vigorous stirring, until the solution became clear. Initially, the dispersed powder in glycerol was yellow in color; then, the solution faded and became colorless, and finally a clear solution without dispersed particles was obtained. At this stage, the glycerol solution was cooled at room temperature; then, a 10% hydrogen peroxide solution (10 mL) was added followed by dropping a sodium hydroxide solution (3 mol/L) until pH = 12. At this point, large dark brown floccules were formed in the solution. Afterwards, this solution was mixed with 60 mL of deionized water and maintained under vigorous stirring for 1 h, at which point the dark brown floccules from the suspension changed into fine dispersed cerium oxide particles of a dark yellow color. The precipitate was washed several times with deionized water and then separated by centrifugation at 10,000 rpm until the pH of the supernatant was neutral. The resulting product was dried at 40 °C for 24 h under vacuum, calcined at 600 °C for 2 h in air, and allowed to cool at room temperature in an oven. The final product was a fine, light-yellow powder of cerium oxide nanoparticles. The CeO_2_ product obtained with sodium hydroxide as precipitating agent was labeled as CeO-2. The synthesis procedure for the sample marked CeO-3 was similar to that for the CeO-2 sample, but the precipitating agent was ammonium hydroxide (10% ammonia solution until pH = 10). Noting that, the same method was applied for the synthesis of the CeO-1 sample, but after adding NaOH as precipitating agent, the reaction mixture was kept at 80 °C for 30 min in order to realize a fast dehydration in the solution of cerium hydroxide.

### 2.3. Characterization

X-ray diffraction (XRD) patterns of samples were performed on a Bruker 18 Avance X-ray diffractometer (Bruker AXS, Karlsruhe, Germany) using CuKα radiation (*λ* = 1.5406 Å), with an accelerating voltage of 40 kV and current of 40 mA, a scanning range between 10 and 120° (2*θ*), a step size of 0.02 degrees/step, and a counting time of 2 s/step. The determination of the XRD parameters was performed using Bruker DIFFRAC-PLUS Evaluation–EVA (ver. 2, Karlsruhe, Germany). Bruker TOPAZ software (ver. 3.0, Karlsruhe, Germany) was applied for data processing: background subtract, peak indexing, crystallite size, and pattern refinement. Diffuse reflectance spectra were recorded on a Shimadzu UV-3600 spectrometer equipped with an integrating sphere, at room temperature. The emission spectra were measured using a Perkin Elmer LS55 spectrometer in a quartz cell of 10 mm path length. The ceria nanoparticles were dispersed in isopropanol. Transmission electron microscopy (TEM) characterization was carried out with a Hitachi High-Tech 4T7700 microscope (Krefeld, Germany) operating at 120 kV. The sample for TEM analysis were prepared by drying nanoparticle dispersion in ethanol on a carbon-coated copper grid. X-ray photoelectron spectroscopy (XPS) measurements were acquired with a Physical Electronic PHI-500 Versa Probe instrument (Chikasaki, Kanagawa, Japan). This system utilizes a monochromatic AlK*α* radiation source (1486.6 eV). The binding energies (BE) were calibrated against the C 1s level (BE = 284.6 eV) of adventitious carbon. Peak deconvolution of the high-resolution XPS spectra was done using PHI MultiPak software (ver. 9.6, Ulvac-PHI, Inc. Chikasaki, Kanagawa, Japan). In order to determine the peak area intensities, the model of background subtraction was iterated Shirley.

### 2.4. Antibacterial Activity

The antibacterial activity of ceria nanoparticles and Ce-doped zinc oxide nanoparticles was examined by the diffusion method, and the minimum inhibitory concentration (MIC) was estimated. Also, the bacterial growth curve in the presence of nanoparticles was determined. The antimicrobial activity of cerium oxide nanoparticles was analyzed and compared with that of cerium-doped zinc oxide nanoparticles prepared in a previous work [22].

#### 2.4.1. Antibacterial Testing

The antibacterial testing of the prepared nanoparticles was performed against Gram-negative (*Escherichia coli* (ATCC 25922)) and Gram-positive (*Staphylococus aureus* (ATCC 25923)) bacteria obtained from the Microbiology Laboratory of the Al. I. Cuza University of Iasi (Microbial Type Culture Collection). The antibacterial effect was tested by the Kirby–Bauer diffusion method [23,24]. The standard bacterial suspension was spread on the surface of Muller–Hinton (MHA) agar plates under sterile conditions, according to MacFarland standards (0.5) in a concentration of 1 × 10^8^ CFU/mL. After the uniform inoculation of the suspension, stainless steel cylinders were applied on the Muller–Hinton solid medium surface using sterile tweezers. Nanoparticle suspension in dimethyl sulfoxide (DMSO) was poured into each cylinder in the following concentrations: 2.5, 5.0, 10.0, and 20.0 mg/mL. Then, the plates were incubated at 37 °C for 24 h. To evaluate the antibacterial activity of the samples, the diameter of the zone of inhibition was measured. Each experiment was performed in triplicate for confirmation. After incubation, zones in which the bacterial growth was inhibited by the antimicrobial sample and zones in which the microorganism was developed due to the lower concentration of the antimicrobial sample, in which its inhibition was not induced, could be observed [25].

#### 2.4.2. Determination of Minimum Inhibitory Concentration

The minimum inhibitory concentration (MIC) means the lowest concentration of an antimicrobial agent that inhibits the growth of a microorganism in the microdilution plates. The MIC was determined for CeO and Ce-doped ZnO nanoparticles by the agar dilution method, which was based on cultures containing different concentrations of ceria nanoparticles in suspension [26]. The determination of the MIC was performed using microplates containing 96 wells, in which 80 μL of MH broth culture medium (MHB), 10 μL of bacterial culture (*Staphylococcus aureus, Escherichia coli*) according to MacFarland standards (0.5). and 100 μL of nanoparticle solutions of different concentrations (20.0, 10.0, 5.0 and 2.5 mg/mL) were introduced. Also, 80 μL of MHB culture medium and 10 μL of bacterial suspension were served as a control sample. After the incubation period at 37 °C for 24 h, 10 μL of resazurin was added in each microwell. The color change from blue–violet to pink (resorufin) showed the growth of microorganisms [27]. The lowest concentration at which no color modification took place was considered the value of the minimum inhibitory concentration of the sample. A significant advantage of resazurin utilization as compared to other metabolic indicators is given by the fact that this compound allows for the continuous monitoring of the cells, because the resazurin does not interfere with the activity of the respiratory chain and is not toxic for cells.

#### 2.4.3. Bacterial Growth Curve

The growth of a bacterial culture over time in the presence of ceria nanoparticles was analyzed using a standard bacterial suspension (*Staphylococcus aureus, Escherichia coli*) that was inoculated in the testing tubes containing 10 mL of MHB medium and 1 mL of nanoparticle suspension. The control sample was MHB culture medium inoculated with the testing microorganism. The absorbance at 600 nm was measured every 3 h for 24 h starting just before incubation. The experiment was repeated three times.

## 3. Results and Discussion

### 3.1. X-ray Diffraction Investigation

**Figure 1:**
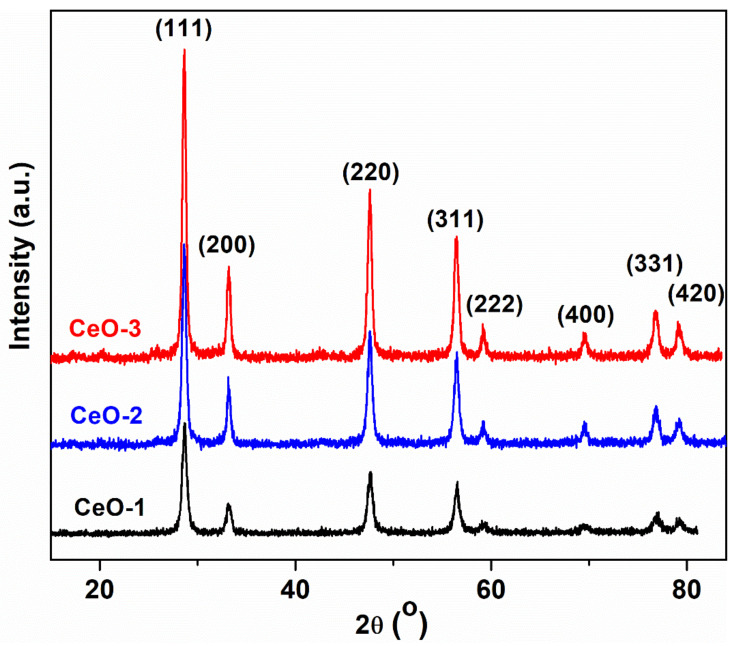
XRD patterns of cerium oxide nanoparticles.

The X-ray diffraction pattern of the synthesized cerium oxide nanoparticles shows very sharp and intense diffraction peaks that demonstrate the high-quality crystalline phase of the nanoparticles and their nanometric size characteristics (Figure 1). Moreover, the diffraction peaks were observed at 2*θ* = 28.61°, 33.15°, 47.57°, 56.45°, 59.14°, 69.53°, 76.18°, and 79.19° (sample CeO-2), corresponding to (111), (200), (220), (311), (222), (400), (331), and (420) crystal planes of the cubic fluorite structure according to the standard JCPDS card No. 34-0394 [28]. The position of the diffraction peaks of the synthetized samples does not significantly differ; all exhibit very sharp peaks, without additional peaks, indicating the absence of cubic structure alteration by impurities or the presence of unwelcome phases during the crystallization process. The main difference between the diffraction signals of the three samples at the same diffraction angle is given by the values of full width at half maximum (FWHM) and peak intensities. When the precipitation was performed at 80 °C, using NaOH as precipitating agent to obtain the CeO-1 sample, the X-ray peaks had lower intensity than that of the CeO-2 sample, where the precipitation took place at room temperature. Changing the precipitation agent, NaOH with NH_4_OH, to prepare CeO-3 at room temperature, the diffraction peaks became sharper and more intense than those of the CeO-1 nanostructure. These differences show the influence of the temperature and the nature of the precipitating agent on the cerium oxide crystal growth. An increased intensity of the diffraction peaks in the order CeO-1 < CeO-2 < CeO-3 indicates an improvement of the crystallinity in the same order [29]. The widening of the diffraction peaks (FWHM) increased in the reverse manner of the peak intensities. This fact is determined mainly by two structural factors that may not be dependent on each other: the crystallite size and internal stress.

The average crystallite size (*D*) was expressed by the Debye–Scherrer equation, *D = 0.9λ/βcosθ*, where *D* represents the average crystallite size, *λ* denotes the X-ray radiation wavelength (CuK*α*: 1.5406 Å), *β* is the full width at half maximum (FWHM) given in radians, and *θ* is the diffracting angle in radians. The crystallite dimension values were calculated for the most intense diffraction peak corresponding to the (111) plane. Using this equation, the calculated crystallite sizes of CeO_2_ nanoparticles are displayed in Table 1 and were found to vary between 13.62 nm (CeO-1) and 17.61 nm (CeO-3). It can be observed that the crystallite size increased when the preparation of cerium oxide nanoparticles was made at room temperature and in the presence of NH_4_OH as precipitating agent instead of NaOH.

Using the Miller indices for the (111) plane, the lattice constant, *a*, can be estimated by the relation (1). The calculated values of the lattice constant are given in Table 1.

(1) $$a=\frac{\lambda \sqrt{3}}{2\sin\theta }$$

Usually, the nanometric dimension of the cerium oxide particles determines an extension of the lattice constant due to the tendency of the network to dissipate the stress caused by the repulsion between the positive charge from the oxygen vacancies and from the Ce^4+^ ions [28]. The presence of the hydrogen peroxide in the system can generate more Ce^3+^ through Ce^4+^ reduction, giving rise to a distortion of the local symmetry and introducing oxygen vacancies due to the oxygen non-stoichiometry [30]. The defects induced by the oxygen vacancies are generally isolated defect types where a positive charge manifests a repulsion action against the surrounding cerium cations, leading to an expansion of the lattice structure. In our case, an opposite effect was found, which consists of lattice contraction due to the decrease of the lattice parameter relating to its bulk value (*a* = 5.4113 Å). This is an unusual result that has not been analyzed in detail in the literature and the existing data are not fully correlated. Chen et al. [31] attributed the lattice contraction effect to the additional atmospheric pressure at the solid–air separation limit. This causes a surface tension leading to the contraction of the lattice as the particle size decreases due to the high surface-to-volume ratio. They set a minimum limit of 15 nm of particle size at which this effect is felt. Below this limit, positively charged oxygen vacancies migrate easily inside the particle, manifesting repulsive forces that lead to lattice expansion. However, in our case, the lattice contraction is beyond this limit, suggesting that the nature of the interactions between oxygen vacancies and nearby metal ions is different. More than that, even earlier than Chen et al., Nachimuthu demonstrated, using X-ray absorption spectroscopy, that at dimensions of CeO_2_ nanoparticles lower than 15 nm, the degradation of the third Ce–O shell can take place with the reduction of the cerium coordination number [32]. This leads to the decreasing of Ce–O and Ce–Ce bond distances with the fluorite cubic crystal structure conservation. Later, Cresi [33], using X-ray absorption fine structure analysis of the CeO_2_ nanoparticles, showed that the Ce–O bond reduction determines lattice contraction at dimensions even smaller than 10 nm. They considered that a low concentration of defects is a favoring factor of lattice compression and the moderate variations of the Ce^3+^ do not significantly influence this effect. In conclusion, the decrease of the lattice parameter is a complex phenomenon that depends on many factors, such as the distribution and nature of oxygen vacancies, other types of defects, or the cerium ion coordination number. Despite these factors, the most important fact is that the lattice contraction does not modify the cubic fluorite structure of the crystal, as can be seen in other studies and in the XRD spectra of our samples.

### 3.2. TEM Analysis

**Figure 2:**
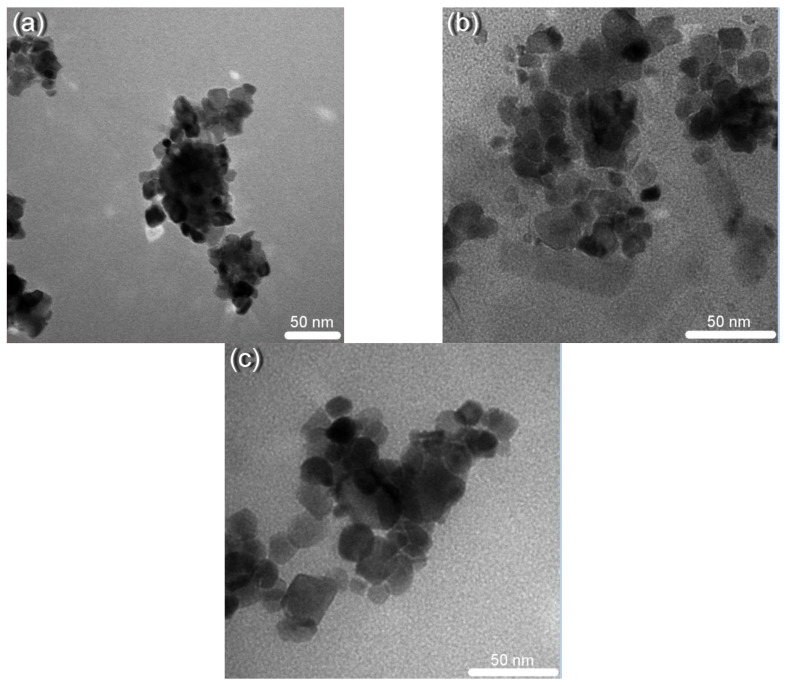
TEM images of cerium oxide nanoparticles: (**a**) CeO-1, (**b**) CeO-2, and (**c**) CeO-3.

The morphology and dimensions of the nanoparticles were studied by TEM microscopy. The TEM images depicted in Figure 2 reveal a non-spherical shape for these nanoparticles. The average particle size was 14.13 nm for CeO-1, 14.21 nm for CeO-2, and 16.53 nm for CeO-3, which is in good agreement with XRD determinations by the Debye–Scherrer model. The small differences of the size values obtained with XRD and TEM, respectively, appear due to fact that XRD diffraction takes under consideration the dislocation cells and TEM only considers the grain particles. Delimitations of the dislocation cells are represented by the low angle boundaries, created by the dislocation wall defects, which diffract X-rays coherently. A grain particle can contain more than one of this type of defect that delimitates dislocation cells, named subgrains. Therefore, the dimensions calculated from the XRD data correspond to the subgrains because the XRD measures the average size of the domains that scatter the X-rays coherently [34]. On the other hand, TEM images are used to measure the size of the entire grain by visual observation. This means the average grain size obtained from TEM images is usually different than the average size of dislocation cells or subgrains as calculated from XRD data.

### 3.3. X-ray Photoelectron Spectroscopy

It is well known that in cerium oxide materials, the metal ion can exist in two oxidation states, +3 and +4, of which Ce^4+^ has the dominant concentration. The reduction of a fraction of Ce from +4 to +3 generates a defective CeO_2-γ_ structure instead of CeO_2_ due to oxygen elimination from the crystal during the reduction reaction:

(2) $${\mathrm{CeO}}_{2}\to {\mathrm{CeO}}_{2-\gamma }+\frac{\gamma }{2}O_{2}$$

**Figure 3:**
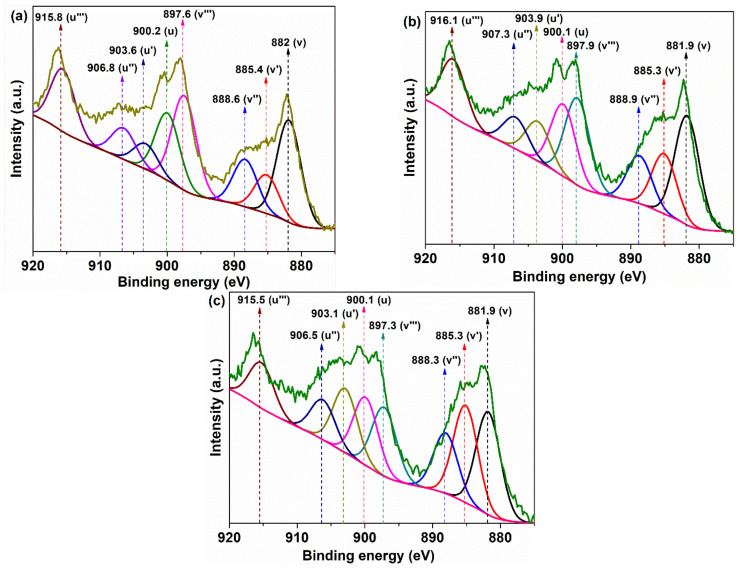
Ce 3d core-level XPS pattern of cerium oxide nanoparticles: (**a**) CeO-1, (**b**) CeO-2, and (**c**) CeO-3.

**Figure 4:**
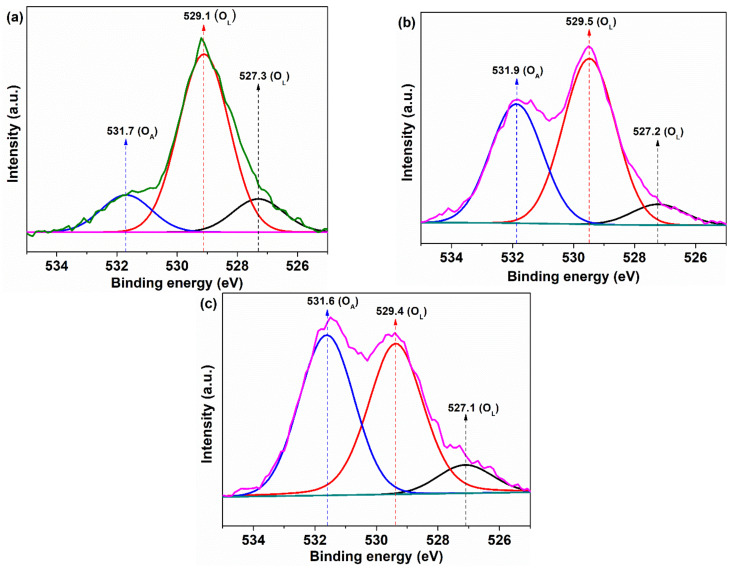
XPS of O 1s core-level of cerium oxide nanoparticles: (**a**) CeO-1, (**b**) CeO-2, and (**c**) CeO-3.

As a result of the removal of oxygen, Ce_2_O_3_ species can appear in the material without preserving the crystalline fluorite structure. On the other hand, the fluorite structure can be preserved, but with the appearance of oxygen vacancies associated with Ce^3+^ ions in the particle’s core or on its surface [35]. This happens when the electrons left in the oxygen vacancies, due to the detachment of the oxygen atoms from the crystal lattice, can reduce Ce^4+^ ions to Ce^3+^. Usually, the small crystallite size associated with the presence of Ce^3+^ induces oxygen vacancies with other structural imperfections, in both the particle’s core and on its surface, generated by the charge imbalance of the two species, Ce^3+^/Ce^4+^. At the surface of the nanoparticle, these defects represent active sites with high free energy that absorb oxygen or other groups based on oxygen atoms, transforming them into species with high reactivity in optimal reaction conditions. Therefore, it is of major importance to determine, at the surface, the concentration of Ce^3+^ ions as well as the absorbed oxygen species, these being an indication of the active sites generated on the surface of the cerium oxide catalyst. XPS measurement was employed to determine these chemical species, recording the core level XPS spectra of Ce 3d and O 1s (Figure 3 and Figure 4). The Ce 3d core level splits in two states by spin–orbit interaction, with Ce 3d_5/2_ and Ce 3d_3/2_ assigned as bands of V and U type, respectively, in the XPS spectrum [36]. The Ce 3d fitted spectra, deconvoluted in eight Gaussian–Lorentzian peaks, were divided into four pairs of doublets noted as (U,V), (U′,V′), (U″,V″), and (U‴,V‴) according to previous works [37,38]. Under these considerations, the four doublets were assigned separately to the two ionic cerium species as follows: U‴, U″, U, V‴, V″, and V being designated to Ce^4+^ ions and the next U′ and V′ being characteristic peaks of Ce^3+^ ions (Figure 3). In the case of the O 1s XPS spectrum, the binding energies lower than 530 eV are usually assigned to oxygen from the lattice of the CeO_2_ crystal, in our case, at around 527 eV and 529 eV (Figure 4). The peak at higher binding energies, in our case around 531 eV, is usually attributed to the weakly absorbed oxygen species on the surface of CeO_2_ associated with Ce^3+^ ions [39,40,41] (Figure 4).

These cover anionic molecules, such as ${\mathrm{OH}}^{-}$ groups, $O_{2}^{2-}$, or neutral molecules, including chemisorbed water. The oxygen deficient cerium oxide is usually characterized by evaluating the ratio of the concentration of oxygen to cerium atoms. There are two ways to calculate this ratio. The first only takes into account the relative concentrations of cerium ions, obtained from the peak areas of the corresponding Ce 3d spectra [42]:

(3) $$\left[{\mathrm{Ce}}^{3+}\right]=\frac{A\left({\mathrm{Ce}}^{3+}\right)}{A\left({\mathrm{Ce}}^{3+}\right)+A\left({\mathrm{Ce}}^{4+}\right)}\times 100\;\left[{\mathrm{Ce}}^{4+}\right]=\frac{A\left({\mathrm{Ce}}^{4+}\right)}{A\left({\mathrm{Ce}}^{3+}\right)+A\left({\mathrm{Ce}}^{4+}\right)}\times 100$$

where $A\left({\mathrm{Ce}}^{3+}\right)$ and $A\left({\mathrm{Ce}}^{4+}\right)$ are the sum of the peaks area corresponding to Ce^3+^ or Ce^4+^, respectively, from the XPS Ce 3d spectrum. The ratio of oxygen to cerium atoms, calculated by taking into account the stoichiometric ratio of O/Ce atoms in Ce_2_O_3_ (3/2) and CeO_2_ (2/1), multiplied by their corresponding Ce^3+^ and Ce^4+^ concentrations, respectively, can be considered the theoretical stoichiometric parameter x:

(4) $$x=\frac{\left[O\right]}{\mathrm{Ce}}=\frac{3}{2}\left[{\mathrm{Ce}}^{3+}\right]+2\left[{\mathrm{Ce}}^{4+}\right]$$

Another method to calculate oxygen per cerium atom ratio takes under consideration the ratio between the sum of the areas from the XPS spectra corresponding to O 1s peaks and those corresponding to Ce 3d. This parameter is noted by *x′* and it represents the actual stoichiometric parameter and takes under consideration the ratio between all concentrations of the oxygen and cerium from the material [41]:

(5) $$x\prime =\frac{O1s}{\mathrm{Ce}3d}=\frac{A_{O}}{A_{\mathrm{Ce}}}\times \frac{S_{\mathrm{Ce}}}{S_{O}}$$

where *A_O_* and *A_Ce_* are the sum of the total XPS integrated area from the peaks corresponding to O 1s and Ce 3d spectra, with their sensitivity factors being *S_O_* = 0.711 and *S_Ce_* = 7.399, respectively. The values of this parameter are considered much closer to reality because it does not take into account the theoretical stoichiometry of the completely oxidized forms of the Ce^4+^ and Ce^3+^ ions, namely, Ce_2_O_3_ and CeO_2_, respectively.

Usually, if the cerium oxide is deficient in oxygen, then x > x′, with values of x lower than 2 and closer to 1.5. Normally, *x′* cannot be higher than the theoretical stoichiometric value x due to the maximum oxygen storage in the lattice for both oxides Ce_2_O_3_ and CeO_2_. The values of the parameters mentioned above are shown in Table 2.

For the samples taken in the study, it is observed that the values of the theoretical stoichiometric parameter, *x*, are smaller than 2 and decrease in the order: CeO-1 > CeO-2 > CeO-3. This trend is a good indicator of the decrease in oxygen content in the same order of the samples. The oxygen deficiency from the lattice can determine the formation of oxygen vacancies. On the other hand, we had the actual stoichiometric parameter x′ < 2 just for CeO-1, with Δx = x − x′ > 0, but for CeO-2 and CeO-3 it was obtained as x’ > 2, with Δx = x − x′ < 0. These are indications of an oxygen surface deficiency for CeO-1 and an excess of oxygen atoms for CeO-2 and CeO-3, exceeding their stoichiometry. The presence of the Ce^3+^ at the surface of nanoparticles causes a charge imbalance and unsaturation with oxygen vacancies formation that facilitate the adsorption or chemisorption of oxygen under different forms, with an unusual coordination [43,44], revealed in O 1s spectra at binding energies higher than 530 eV (Figure 4a). In such a case, the oxygen content from cerium oxide nanoparticles can exceed its stoichiometry. Oxygen adsorption at the surface level indicates a highly active surface, generated by the presence of oxygen vacancies, which makes the nanoparticle suitable for catalytic processes [39]. The percentage of adsorbed oxygen ([O_A_]) relative to the total amount of oxygen was calculated using the area of the peaks corresponding to the lattice oxygen (*A*(O_L_)) and adsorbed oxygen (*A*(O_A_)) from O 1s spectra according to the following formula:

(6) $$\left[O_{A}\right]=\frac{A\left(O_{A}\right)}{A\left(O_{A}\right)+A\left(O_{L}\right)}\times 100$$

where *A*(O_L_) represents the sum of the two-peak area with the maximum at binding energies less than 530 nm, assigned to lattice oxygen [43,44], and *A*(O_A_) is the peak area with the maximum at binding energies higher than 530 eV, assigned to adsorbed oxygen, listed in Table 2.

The values of the percentage of adsorbed oxygen are given in Table 2 and reveal that the [O_A_] increases in the order CeO-1 < CeO-2 < CeO-3. Moreover, the concentration of Ce^3+^ increases in the same direction with the percentage of adsorbed oxygen, suggesting that the Ce^3+^ ion favors the superficial oxygen adsorption. It is known that an increased content of Ce^3+^ ions, generated by Ce^4+^ reduction, determines the appearance of oxygen vacancies, necessary for keeping the charge balance, as in the following reaction represented in Kroger–Vink notation [45]:

(7) $$4{\mathrm{Ce}}_{\mathrm{Ce}}+O_{O}\overset{}{\to }2{\mathrm{Ce}}_{\mathrm{Ce}}+2{\mathrm{Ce}}_{\mathrm{Ce}}^{\prime }+V_{O}^{˙˙}+\frac{1}{2}O_{2}\left(g\right)$$

Therefore, there is a good correlation between [O_A_] and Ce^3+^, which suggests that the oxygen is adsorbed on the surface of the nanoparticles at the level of oxygen vacancies generated by the presence of superficial Ce^3+^ ions. Holgado et al. [43] found in their studies that this common trend is a result of the fact that the peak from O 1s spectra that correspond to adsorbed oxygen may by related mainly to the cerium oxide from the exposed surface. In this interpretation, our results suggest that, compared to the other two samples, in the CeO-1 sample, from the total oxygen vacancies, a smaller fraction is on the exposed surface of the nanoparticles, leading to a smaller percentage of adsorbed oxygen. Theoretically, this makes the surface of the CeO-2 and CeO-3 samples, which have a high percentage of adsorbed oxygen, much more active than that of the CeO-1 sample.

### 3.4. Optical Properties

**Figure 5:**
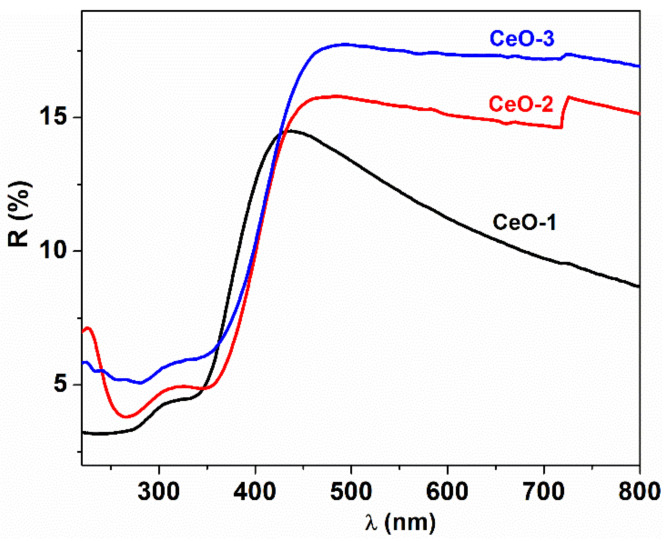
UV-Vis diffuse reflectance spectra of CeO_2_ nanoparticles.

The evaluation of the gap energy was done using diffuse reflectance spectra of the as-synthesized cerium oxide nanoparticles deposited on the quartz substrate as a film (Figure 5). The reflectance data were processed within the Tauc model equation, where the Kubelka–Munk function was introduced [46]:

(8) $${\left[F\left(R_{\infty }\right)E\right]}^{1/n}=B\left(E-E_{g}\right)$$

with $F\left(R_{\infty }\right)=\left(1-R_{\infty }\right)/R_{\infty }$ defined as the Kubelka–Munk function; $R_{\infty }=R_{s}/R_{Std}$, where *R_s_* and *R_std_* are the reflectance of the sample and standard, respectively; and *E = hν*, where *E_g_* denotes the gap energy and *n* is a parameter that takes values depending on the type of transition. Usually, the calculation of *E_g_* is made by evaluating the linearity of the equation (1) for the two types of transition: allowed direct (*n* = ½) and allowed indirect (*n* = 2).

**Figure 6:**
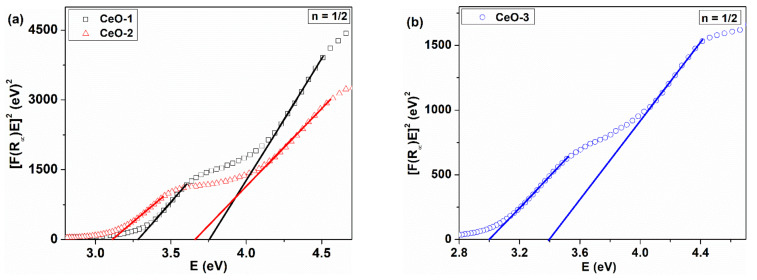
*[F(R)hν]^2^* versus *hν* plots of CeO_2_ nanoparticles: (**a**) CeO-1 and CeO-2, (**b**) CeO-3.

**Figure 7:**
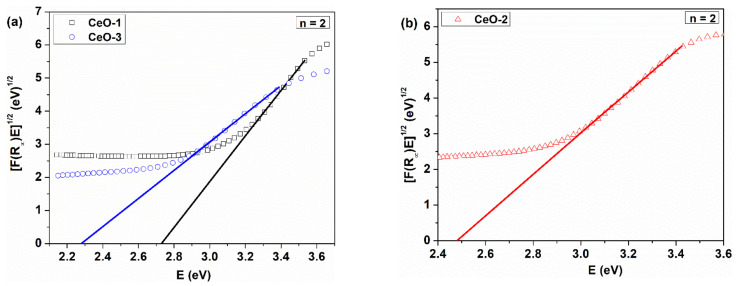
Tauc plots of *[F(R)hν]^1/2^* as a function of energy for cerium oxide nanoparticles: (**a**) CeO-1 and CeO-3, (**b**) CeO-2.

The plots of *[F(R)hν*]^1/n^ as a function of photon energy are displayed for the direct and indirect allowed transitions in Figure 6 and Figure 7. The representation of the Tauc equation modified with the Kubelka–Munk function reveals two energy gaps for the direct and one for the indirect allowed transition. In the literature, the energy of the forbidden band, calculated from reflectance measurements, is 6 eV, but the values obtained here are below this limit [47]. The valence band is formed mainly by 2p orbitals coming from oxygen atoms, noted as O 2p, and the conduction band, Ce 5d, is constructed by the 5d orbitals from cerium. The 4f atomic-like orbitals originated from cerium atoms are situated between these bands as a narrow extended state localized in the forbidden zone. The transition O 2p→Ce 4f is attributed to both direct and indirect transition [48]. This explains the smaller value of the obtained gap energy as compared to the width of the forbidden band [49].

XPS measurements showed that cerium oxide nanoparticles are CeO_2-γ_ compounds, where Ce^3+^ and Ce^4+^ coexist under different proportions. Skorodumova et al. [50] demonstrated that the nature of bonding in cerium oxide is polarized ionic and the 4f electrons present in Ce_2_O_3_ do not participate in the bonding. More than that, the unoccupied 4f orbital originating from CeO_2_ is an empty atomic-like level. The presence of the two possible oxidation states of cerium, Ce^3+^/Ce^4+^, determines an imbalance of stoichiometry with a disturbance of the electrical neutrality in the crystal lattice. The presence of the two possible oxidation states of cerium, Ce^3+^/Ce^4+^, determines an imbalance of stoichiometry with a disturbance of the electrical neutrality, generating oxygen vacancies and local distortion of the CeO_2_ lattice. In this case, the Ce 4f band splits into two localized states in the forbidden zone. The first state, on the energy scale, is partially occupied with electrons, named 4f_full_, and the second is an unoccupied 4f state, named 4f_empty_, with a higher energy than the former [51]. The exact position of the localized states in the forbidden band induced by the oxygen vacancies and other structural defects could not be known, but these states can explain why there is more gap energy for the direct transition. There are separate transitions which may imply these two types of 4f localized states. Patsalas et al. [52] assign the O 2p–Ce 4f direct transition exclusively to the CeO_2_ structure, despite the violation of the angular momentum selection rule. This mismatch is counteracted by the strong d–f characteristic of the transition due to the presence of Ce 4d electrons in the valence band. The same situation was mentioned by Calvache-Muñoz et al. [53], where two direct band gap energies, higher than 3 eV, were found. The highest gap energy (3.9 eV) was assigned to the O^2−^−Ce^4+^ charge transfer due to the O 2p–Ce 4f transition and the smallest gap energy (3.4 eV) was assigned to the f–d electronic transition in the Ce^3+^ ions, which is present in our defective CeO_2-γ_ structure, as we mentioned in the XPS analysis. Our values of the direct transition energies are lower than the abovementioned values, but they are in the range of values indicated in the literature [54].

As can be seem from Table 3, there is a red shift of the gap energy for the direct transition, with the *E_g_* values decreasing in the following order: CeO-1 > CeO-2 > CeO-3 with increasing Ce^3+^ content. The gap energy for indirect transition corresponds to the O 2p–Ce 4f electronic transition and it is considered a fundamental transition exclusively for the CeO_2_ type oxide [52]. Also, in this case we recorded a red shift of the gap energy with increasing Ce^3+^ content, as was mentioned other reports [55]. Patsalas et al., in their ellipsometric study, ascribed this process to the accumulation of the Ce^3+^ at the grain boundaries [52]. The presence of Ce^3+^ ions could generate oxygen vacancies or other types of defects that determine a red shift of the gap energies [56]. Such results are in agreement with our XPS measurements and interpretation, where the Ce^3+^ concentration increases in the order of CeO-1 < CeO2 < CeO-3 (Table 3), leading to an increased number of oxygen vacancies or other types of defects; in this case, the CeO-3 sample has the greatest number of defects.

Near the fundamental absorption edge, in crystals that have regions with disturbed order, the optical absorption depends exponentially on the energy of the absorbed radiation, according to the Urbach rule [57,58]:

(9) $$\alpha =\alpha_{0}e^{E/E_{U}}$$

where *α_0_* is a constant, α is the optical absorption coefficient, E denotes the photon energy, and *E_U_* represents the Urbach energy (the transition energy between the fundamental absorption edge and the tail states localized near the absorption edge in band gap). The width of these localized states is determined by the structural and morphological defects that appear in the crystalline materials. Therefore, *E_U_* can be interpreted as a disorder parameter in the crystal lattice. To have some information about the degree of disorder in our samples, from diffuse reflectance data, the modification of Equation (9) with the Kubelka–Munk function has been made [22] and the linearized form becomes the following:

(10) $$lnF\left(R_{\infty }\right)=ln\beta +\frac{E}{E_{U}}$$

where *β* is a constant.

**Figure 8:**
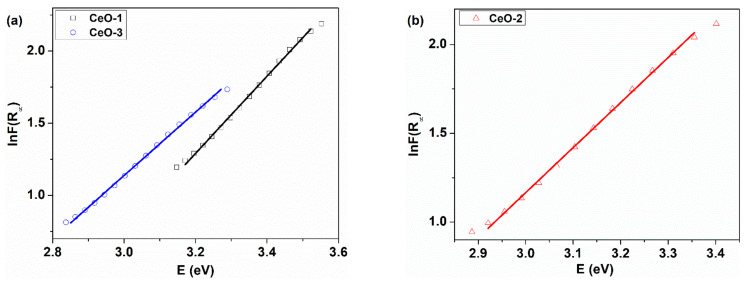
Urbach energy curves for cerium oxide nanoparticles: (**a**) CeO-1 and CeO-3, (**b**) CeO-2.

The graphical representation of Equation (10) allows *E_U_* to be calculated from the reverse of the slope (Figure 8). The value of Urbach energy reveals an increasing trend from 374.3 meV for CeO-1 to 394.45 meV for CeO-2 and 455.38 meV for CeO-3 (Table 3). The increase of the width of localized state tails is also well correlated with an increased content of Ce^3+^ from CeO-1 to CeO-2 and CeO-3. These results suggest the contribution of Ce^3+^ to the creation of oxygen vacancies or other defects, leading to the appearance of local disorder in the crystalline lattice. These things generate defective structures such as CeO_2-γ_, depending on the synthesis conditions, such as temperature regime or precipitating agent.

### 3.5. Fluorescence Spectra

**Figure 9:**
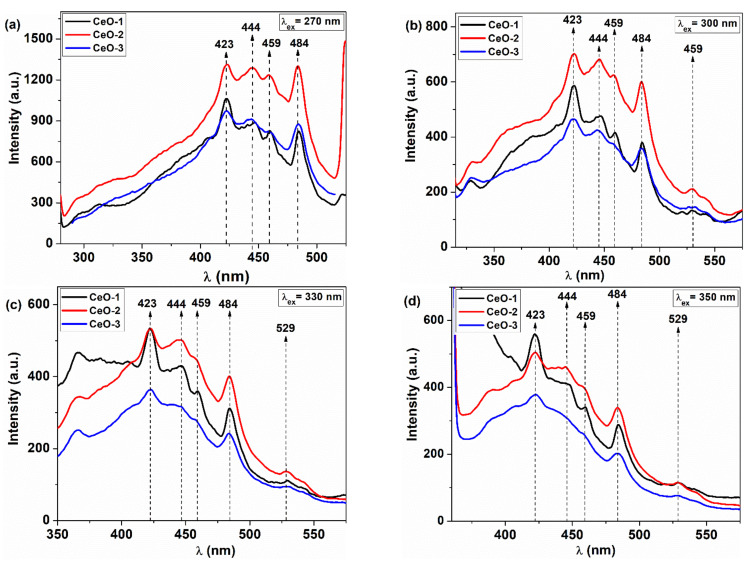
Fluorescence spectra of cerium oxide nanoparticles for different excitation wavelengths: (**a**) 270 nm, (**b**) 300 nm, (**c**) 330 nm, (**d**) 350 nm.

The photoluminescence spectra of cerium oxide nanoparticles are depicted in Figure 9 at different excitation wavelengths, namely, 270, 300, 330, and 350 nm, respectively. All samples present three blue emission bands around 422, 444, and 459 nm, one blue–green emission at 484 nm, and a band located at 529 nm belonging to green emission. However, the presence of the near band emission is not clearly visible for the cerium oxide samples. Mochizuki et al. showed that this involves a radiative recombination of an exciton with significant Coulomb interaction, in which the electron is located around the Ce^4+^ ion within a complex formed between cerium ions and oxygen vacancies [59]. Increasing the excitation wavelength, the emission intensity becomes lower due to the decrease of the penetration depth, leading to a low fluorescence response, but the position of the emission maxima does not practically change for these samples. These results are in good agreement with other data reported for cerium oxide nanoparticles [28,60]. The emission bands between 350 and 550 nm can be associated with the presence of the electronic states localized between O 2p and Ce 4f levels that are generated by structural defects, including oxygen vacancies [60,61]. It is worth noting that the strongest emission was observed for the CeO-2 sample at *λ_ex_* = 270 nm (Figure 9a) and the emission intensity of the CeO-1 and CeO-3 samples are comparable, but lower than that of the CeO-2 sample at the same excitation wavelength. Therefore, similar radiative decay paths are accessed at this excitation wavelength for the CeO-1 and CeO-3 samples. More than that, the reduced emission intensity for CeO-1 and CeO-3 suggests the presence of different defective electronic states that act as electron traps with a non-radiative energy dissipative effect, lowering the fluorescence intensity. The increase of the excitation wavelength determines a nonlinear variation of fluorescence intensity. The emission intensity of CeO-1 becomes higher than that of CeO-3 as can be seen in Figure 9b,c and even higher than that of CeO-2 sample for the blue emission at 423 nm (Figure 9d). This is a result of different sets of defects generated by different routes of nanoparticle synthesis. Their corresponding defective states give the possibility of many decay paths with different radiative emission efficiencies. Depending on the excitation wavelength, different isolated states are accessed with different decay paths, leading to the modification of emission band intensities. Among all three samples, CeO-3 appears to retain the lowest intensity in the emission spectrum regardless of the excitation wavelength. As mentioned before, the high ratio of Ce^3+^/Ce^4+^ can generate different types of defects, creating more disorder in the lattice and increasing the Urbach energy. This gives the possibility of an efficient transfer of energy between neighboring states to the defective states that act as quenching sites, leading to low fluorescence intensity. In the case of the CeO-1 and CeO-2 samples, there is no clear correlation between lattice disorder and fluorescence intensity, but it seems to exist in the case of the CeO-3 sample, where the highest values were obtained for the Ce^3+^/Ce^4+^ ratio and Urbach energy, leading to the lowest fluorescence intensity. On the other hand, these non-radiative centers, like electron trapping sites, enhance the possibility of the catalytic applications for CeO-3 [62].

### 3.6. Antibacterial Activity

Cerium oxide nanoparticles have been reported to present a broad range of Gram-positive and Gram-negative bacteria [63,64]. The evaluation of the antibacterial activity of cerium oxide nanoparticles was studied against both pathogenic Gram-positive and Gram-negative bacteria using four different concentrations of nanoparticles (2.5, 5.0, 10.0, and 20.0 mg/mL). The results for the antibacterial activity of the CeO-1 sample against Gram-positive *Staphylococcus aureus* indicates that the microorganism growth was inhibited at the following concentrations: 10 and 20 mg/mL, respectively, with the diameter of the zone of inhibition (ZOI) being 6 and 9 mm, respectively (Table 4). Also, it is observed that the growth inhibition for *Escherichia coli* occurred for the same concentrations of nanoparticles, but a slightly better effect of inhibition was found here (Table 4). The sample containing 20.0 mg/mL of CeO-1 exhibited a higher antibacterial effect with the diameter of the zone of inhibition being around 11 mm (Table 4) against *Escherichia coli*. It was noticed that the diameter of the zone of inhibition increased with the increase in cerium oxide content.

The antimicrobial patterns of CeO-3 sample were higher against *Staphylococcus aureus* and *Escherichia coli* as compared to those of the CeO-1 sample (Table 4). The good antibacterial activity of the CeO-3 sample towards the tested bacteria was evidenced by the increase of the diameter of the zone of inhibition which increased as the nanoparticle concentration became higher (Table 4). However, the diameters of the zone of inhibition were higher for Gram-positive bacteria than those for the Gram-negative bacteria (Table 4). The enhanced antibacterial activity of the CeO-3 sample can be related to the nanoparticle size. Thus, the nanoparticles with higher crystallite size (17.61 nm for CeO-3) present antimicrobial activity at lower concentrations as compared to the CeO-1 sample with lower crystallite size (13.62 nm) (Table 1). Also, the better antimicrobial effect of CeO-3 nanoparticles can be due to the increased content of adsorbed oxygen on the surface nanoparticles (Table 2) according to the XPS measurements. It can be seen that the Gram-positive bacteria were more susceptible to the CeO-3 nanoparticles as compared to the Gram-negative bacteria due to the different structure of the cellular walls and the interaction of the cerium ions with the bacterial cell wall leading to an easy penetration of the cell membrane by cerium oxide nanoparticles [65]. The results obtained in this work are in agreement with previous studies on the antimicrobial activity of cerium oxide nanoparticles. Thus, green prepared cerium oxide nanoparticles showed a significant effect against (G+) and (G−) bacteria at concentrations of nanoparticles of 50 or 100 mg/mL [66,67]. Similarly, cerium oxide nanoparticles obtained by a chemical method have been shown to have growth inhibition against *Escherichia coli* depending on the nanoparticle concentration in the range of 50–100 mg/mL [68]. The agar diffusion method has frequently been utilized to evaluate the susceptibility of *Staphylococcus aureus* against cerium oxide nanoparticles. It was found that in some cases cerium oxide nanoparticles had a weak antibacterial activity [69], but in other cases the cerium oxide nanoparticles efficiently prevented the growth of this bacterial pathogen [70].

The antibacterial activity of cerium oxide-doped zinc oxide nanoparticles was examined in the same range of concentrations against Gram-positive *Staphylococcus aureus* and Gram-negative *Escherichia coli* bacteria. The results of the antibacterial activity indicated that the Ce-doped ZnO nanoparticles (ZnCeO) presented an inhibition effect at a concentration of 20 mg/mL, the diameter of the zone of inhibition being 11 nm against *Staphylococcus aureus*. These nanoparticles did not show a zone of inhibition towards *Escherichia coli* at all the tested concentrations (Table 4). Relatively few studies have been reported about the effect of cerium-doped zinc oxide nanoparticles on bacterial systems [71]. However, Ce-doped ZnO thin films displayed antibacterial activity against Gram-positive *Staphylococcus aureus* and Gram-negative *Pseudomonas aeruginosa* bacteria at concentrations of 1, 2, 4, and 8 mg/mL, giving large zones of inhibition at higher concentrations [71].

Furthermore, the antibacterial activity of cerium oxide nanoparticles was determined by the microdilution approach using resazurin as colorimetric indicator of cell viability and the corresponding minimum inhibitory concentration values were estimated towards the same microbial pathogens at different concentrations (2.5, 5.0, 10.0, and 20.0 mg/mL). The cerium oxide nanoparticles revealed significant inhibitory activity against all the tested pathogens even at relatively low concentrations (Table 5). In this case, the CeO-1 and CeO-3 samples inhibit the growth of bacterial strains at an MIC value of 2.5 mg/mL for *Staphylococcus aureus* and 5 mg/mL for *Escherichia coli*, whereas cerium-doped zinc oxide nanoparticles required a higher MIC value to inhibit the bacterial growth of the tested microorganisms (20 mg/mL). The ability of cerium oxide nanoparticles to induce inhibitory effects towards some microbial strains has been pointed out in the literature. MIC values of 2.15 and 10 mg/mL were found for *Escherichia coli* and *Staphylococcus aureus* [65], respectively, or 50 mg/mL for *Staphylococcus aureus* and 100 mg/mL for *Escherichia coli* [72].

**Figure 10:**
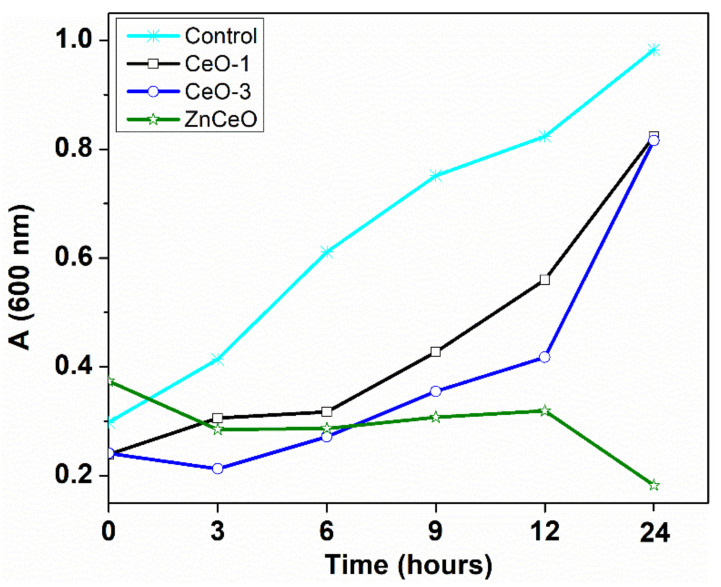
Dynamic growth curves for *Staphylococcus aureus* treated with cerium oxide nanoparticles.

The growth inhibition pattern was performed for the two different bacterial strains. The growth of bacteria *Staphylococcus aureus* in the presence of CeO-1 sample (2.5 mg/mL) was depicted in Figure 10. An increase of the absorbance at 600 nm after 6 h from incubation towards *Staphylococcus aureus* was observed, followed by a progressive increase until 24 h, which is the exponential phase growth. The lag-phase growth occurs in the first 3 h of incubation. The growth of *Staphylococcus aureus* in the presence of the CeO-3 sample occurs after 3 h from incubation and progressively increases until 24 h, and was also found to be the exponential phase growth (Figure 10). The effect of the cerium oxide nanoparticles (CeO-1, CeO-3) on the growth of the tested microorganisms was practically the same, because the same value of the absorbance was recorded after 24 h (Figure 10). A much stronger growth inhibition of the growth of *Staphylococcus aureus* was observed in the presence of Ce-doped zinc oxide nanoparticles (Figure 10). The *Staphylococcus aureus* culture presented a stationary phase between 3 and 12 h, when a relatively constant number of bacterial cells were present, and then between 12 and 24 h the number of bacterial cells decreased (decline phase) with an absorbance value of 0.19 at 24 h (Figure 10).

**Figure 11:**
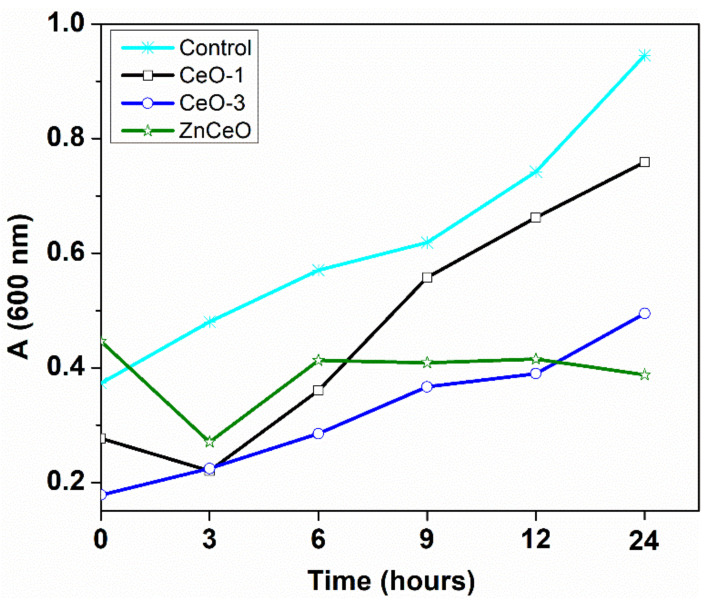
Growth curves of *Escherichia coli* treated with cerium oxide nanoparticles.

The effect of cerium oxide nanoparticles on the growth of *Escherichia coli* was depicted in Figure 11. It is worth mentioning that the bacterial growth of *Escherichia coli* treated with CeO-3 nanoparticles was more strongly inhibited (Figure 11), as compared to that of the CeO-1 sample, where a lower inhibition activity was found with a value of absorbance of 0.76 relating to the value of 0.49 for CeO-3. An increase of absorbance occurred after 3 h from incubation and then a progressive increase took place until 24 h, which can be considered as the exponential growth phase. The growth of *Escherichia coli* in the presence of CeO-3 occurred between 0–9 h, followed by the stationary phase between 9–12 h and a slight progressive growth until 24 h. Similarly, the antimicrobial pattern of Ce-doped ZnO nanoparticles can be described by a decrease of absorbance in the first 3 h. Afterwards a progressive increase of the absorbance occurred after 3 h (the exponential growth phase); then, after 6 h a stationary phase was observed and the decline phase appeared when the number of bacterial cells decreased. The highest inhibition effect for *Escherichia coli* was determined by Ce-doped zone nanoparticles (20 mg/mL), the absorbance being 0.39 after 24 h, which was lower compared to the value of the control sample (Figure 11).

## 4. Conclusions

In this paper, a new route of cerium oxide nanoparticles synthesis by the precipitation method has been reported, using glycerol as the dispersion medium for cerium sulphate, a precursor that is almost insoluble in water at neutral pH. The impact of synthesis parameters on the structure, morphology, and optical properties of nanoparticles with their antibacterial applications was studied. The XRD analysis of cerium oxide nanoparticles revealed network contraction regardless of the synthesis parameters, with the network constant being smaller than its corresponding bulk value. Morphological analysis indicated dimensions of cerium oxide nanoparticles less than 20 nm, the smallest dimensions being found in the case of the sample obtained by precipitation with NaOH at 80 °C. Defective oxygen structures such as CeO_2-γ_ were found using XPS measurements, with higher surface activity in the case of nanoparticles obtained by precipitation at room temperature or using NH_4_OH as precipitating agent instead of NaOH. The study of electronic transitions highlighted the presence of two thresholds of absorption for direct allowed transition with a red shift of their corresponding gap energies, in good correlation with the increasing content of Ce^3+^ ions. Interband localized electronic states generated by defects were confirmed from reflectance spectra through Urbach energy calculation and emission spectra in the visible range. The good antibacterial activity of cerium oxide nanoparticles may be due to the surface Ce^3+^ and the presence of oxygen vacancies.

## Figures and Tables

**Table 1:** XRD parameters of cerium oxide nanoparticles.

Sample	T (°C)	Precipitating Agent	D (nm)	a (Å)
CeO-1	80	NaOH	13.62	5.3691
CeO-2	25	NaOH	15.48	5.3802
CeO-3	25	NH_4_OH	17.61	5.3765

**Table 2:** Summary of XPS parameters of cerium oxide nanoparticles.

Sample	x	x′	Δx = x − x′	[Ce^3+^] (%)	[O_A_] (%)
CeO-1	1.93	1.4	0.53	14.69	15.01
CeO-2	1.89	2.17	−0.28	21.02	39.38
CeO-3	1.85	2.3	−0.45	30.55	47.04

**Table 3:** Parameters extracted from Tauc and Urbach equations for cerium oxide nanoparticles.

Sample	Synthesis Conditions	E_d1_ * (eV)	E_d2_ * (eV)	E_i_ ** (eV)	E_U_ (meV)
CeO-1	80 °C, NaOH	3.28	3.76	2.72	374.30
CeO-2	25 °C, NaOH	3.10	3.66	2.47	394.45
CeO-3	25 °C, NH_4_OH	3.00	3.39	2.28	455.38

* Direct allowed transition energy (*n* = 1/2); ** Indirect allowed transition energy (*n* = 2).

**Table 4:** Antimicrobial activity of cerium oxide nanoparticles against bacterial pathogens.

Sample	Concentration (mg/mL)	Diameter of Zone of Inhibition (mm)	
		*S. aureus*	*E. coli*
CeO-1	2.5	0	0
	5.0	0	0
	10.0	6	10
	20.0	9	11
CeO-3	2.5	0	0
	5.0	10	0
	10.0	11	10
	20.0	13	12
ZnCeO	2.5	0	0
	5.0	0	0
	10.0	0	0
	20.0	11	0

**Table 5:** Minimal inhibitory concentration (MIC) (mg/mL) of the cerium oxide nanoparticles against tested microorganisms.

Sample	Bacterial Strain	
	*S. aureus*	*E. coli*
CeO-1	2.5	5.0
CeO-3	2.5	5.0
CeOZn	20.0	20.0

## Data Availability

Data are available from the corresponding author upon reasonable request.
